# Perception of fertility, quality of life, and depression in women undergoing assisted reproductive treatment

**DOI:** 10.5935/1518-0557.20240103

**Published:** 2025

**Authors:** Gisleine Verlang Lourenço, Vania Naomi Hirakata, Paula Barros Terraciano, Pietra Giron, Tania Marques, Eduardo Pandolfi Passos

**Affiliations:** 1 Clinical Psychologist/FABIN, Porto Alegre, Brazil; 2 PPGGO/UFRGS, Porto Alegre, Brazil; 3 Unidade de Bioestatística-HCPA, Porto Alegre, Brazil; 4 Laboratório de Embriologia e Diferenciação Celular-CPE/HCPA, Porto Alegre, Brazil; 5 Acadêmica da Medicina UFPEL, Pelotas, Brazil; 6 FACED/UFRGS, Porto Alegre, Brazil; 7 Centro de Fertilidade/Hospital Moinhos de Vento, Porto Alegre, Brazil

**Keywords:** Infertility, Quality of life, Depression

## Abstract

**Objective:**

To investigate perception of (in)fertility, fertility-related quality of life, and depression in women undergoing assisted reproductive treatment.

**Methods:**

Cross-sectional study, quantitative approach. The research sample comprised 89 women participating in the assisted reproduction program at the Hospital de Clínicas de Porto Alegre (HCPA) outpatient clinic. Data collection took place between August 2016 and January 2018. The tools used in the study were the Fertility Quality of Life (FertiQoL) questionnaire, Fertility Problem Inventory (FPI), Beck Depression Inventory (BDI), and a questionnaire on sociodemographic data.

**Results:**

The mean total FertiQoL score was 66.5 ± 14.5, and it was significantly associated with depression and formal education; on average, patients with depression had a score difference of -10.7 (95CI%: -17.5;-3.8) compared to those without depression. Patients with depression reported a lower quality of life compared to those without depression in the social, treatment environment, and total treatment subscales. On the mind/body subscale, those meeting BDI criteria for depression scored 13.4 points lower on average than respondents without depression (*p*<0.001). The highest-scoring FPI dimension was conjugal and sexual relationship (4.5±0.79). The FPI dimensions social relationships (r= -0.77; *p*<0.01), conjugal and sexual relationship (r= 0.67; *p*<0.01), and maternity/paternity (r= -0.65; *p*<0.01) correlated with FertiQoL total score.

**Conclusions:**

Women with depression who are in assisted reproductive treatment endorse lower fertility-related quality of life than their peers without depression. Assisted reproduction providers should be aware of the multiple factors involved and offer psychosocial care before, during, and after treatment.

## INTRODUCTION

According to the World Health Organization, infertility is a disease that affects nearly 15% of couples who are trying to get pregnant. It affects from 50 million to 80 million people worldwide and nearly 8 million people in Brazil (WHO, 2023). Its causes may be ranked into four groups: (1) tubo-peritoneal factors, related to endometriosis and sequelae of pelvic inflammatory disease; (2) male factors, i.e., abnormalities in the number, motility, and morphology of sperm cells; (3) hormonal factors, such as ovulation disorders, polycystic ovary syndrome, and abnormalities in thyroid hormones and prolactin levels; and (4) unknown factors, i.e., which remain unidentified after a thorough workup (Passos *et al.*,., 2007).

Assisted reproduction techniques, which serve not only for the treatment of infertility but also propose a new form of reproduction, include in vitro fertilization (IVF), artificial insemination (AI), and intracytoplasmic sperm injection (ICSI). Two other modalities, less frequent in the Brazilian context, are gamete donation and surrogacy (Ferriani & Navarro, 2004). In 1978, the first child conceived via IVF was born in England, with a natural cycle performed by Dr. Robert Edwards and Dr. Patrick Steptoe; in Brazil, the first assisted reproductive procedure took place in 1984. By and large, recourse to IVF is only had when artificial insemination is not indicated; however, in cases of advanced age or severe male factors, it is indicated as a first-line treatment (Passos *et al*., 2007). Sterility is defined by failure to produce viable gametes (eggs and sperm) or zygotes (the result of egg and sperm fusion). Excepting situations where there is a physiological or anatomical alteration determining permanent childbearing incapacity, a couple’s inability to conceive may be due to causes that are attributable to both partners and might even not be a problem if those individuals intended to have children with another genitor. Thus, a couple is deemed *infertile* when its odds of conceiving are reduced, but this can be circumvented by medical measures (e.g., oligospermia), and *sterile* when the couple’s natural childbearing ability is nil-e.g., when a woman has both tubes obstructed and her husband has no sperm in his ejaculate (azoospermia). Hence, it is advisable that both spouses attend the initial fertility visit, so further investigation of implied causes can be performed in an integrated fashion. The need for additional tests is based on the outcomes of this preliminary investigation (Santos & Moura, 2010).

The association between quality of life and infertility has been widely studied (Chachamovich *et al*., 2010a; Chachamovich *et al*., 2010b; Chachamovich *et al*.,., 2010c; Chachamovich *et al*., 2009). Infertility has been described as a source of anxiety for most couples who are affected by it (Lourenço, 2010; Gourounti, 2016).

In the past, several generic measurement tools were used to evaluate QoL among *infertile* patients. More recently, however, a specific QoL measurement tool for infertile couples was designed and has been used internationally: the *Fertility quality of life tool* (FertiQoL) (Boivin et al., 2011; Hsu, *et al*., 2011; Aarts *et al*.; 2011).

IVF has been described as a multidimensional stressor, as it involves both features pertaining to the experience of infertility as well as those related to the treatment itself (Verhaak *et al*., 2007). Although male partners often experience embarrassment caused by issues related to “manhood” or semen collection, women suffer the bulk of the treatment demand, being subjected to countless invasive tests, having to follow strict drug prescriptions, and receiving high doses of hormones. In this context, it is hard to tell the impact of infertility apart from that of treatment (Eugster & Vingerhoets, 1999; Chachamovich *et al*., 2010a).

Several countries have issued guidelines recommending that psychological care be provided at reproductive centers (Dìaz, 2007; Espada & Moreno-Rosset, 2008; Moreno-Rosset *et al*.,2009; Gameiro *et al*., 2015; Anzica, 2016; Asrm, 2017; Sánchez, 2017). In Brazil, the Brazilian Society of Assisted Reproduction (SBRA) published in 2012 the 1st Consensus on Psychology in Assisted Reproduction, and a 2017 Federal Medical Council directive (updated in 2022) proposed recommendations regarding the scope of practice of psychologists in this context. In 2018, these recommendations were compiled into a textbook (*Guia de Recomendações de Atenção Psicossocial nos Centros de Reprodução Assistida*; Straube *et al*., 2018).

The aim of this study is to investigate the perception of infertility, quality of life, and depression in women undergoing assisted reproduction treatment. Considering the context described above, we highlight the importance of identifying emotional factors in infertile women so that interventions can be designed and proposed to help preserve their quality of life.

## METHODS

A quantitative-based cross-sectional study was performed. The sample included 89 participants who were enrolled in the assisted reproduction program of the Hospital de Clínicas de Porto Alegre (HCPA) outpatient fertility clinic. Data collection extended from August 2016 to January 2018.

All women undergoing assisted reproduction treatment at the HCPA clinic during this period were eligible. The inclusion criteria were age 35 years or younger, no previous explicit diagnosis of any mental disorder, and no previous diagnosis of organic diseases. Those who met the criteria agreed to take part in the study, and provided written informed consent were included in the sample. The instruments were administered to those who agreed to participate at the time and place of their appointments, individually or in groups. Tools were administered in the following sequence: sociodemographic data questionnaire > Fertility FertiQoL (Boivin *et al*., 2011) > Beck Depression Inventory (BDI) (Beck & Steer, 1996) > Fertility Problem Inventory (FPI) (Newton *et al*., 1999). All were self-administered in the presence of psychology students and trained psychologists. This study was approved by the HCPA Research Ethics Committee (Certificate of Submission for Ethical Approval: 57125816.3.0000.5327).

### Tools

#### Sociodemographic data questionnaire

The questionnaire used to characterize the sample included items on age, ethnicity, place of birth, educational attainment, duration of relationship, length and cause of infertility, fertility treatments received to date, previous children, and data on the partner.

#### FertiQOL

FertiQoL, an internationally developed and validated questionnaire, is composed of two general items and two QoL measurement modules (Core FertiQoL and the optional Treatment FertiQoL). The Core FertiQoL includes 36 items divided into four subscales: Emotional, Mind-Body, Relational, and Social. The optional Treatment module evaluates QoL related to fertility treatment per se, covering Environment and Tolerability. In this study, the Brazilian version of both modules, with five answer categories, will be used. A higher score in the total FertiQoL scale or in any one of its subscales denotes better QoL (Boivin *et al*., 2011).

#### BDI

The Beck Depression Inventory is a research and clinical tool used worldwide to detect depressive symptoms. It is composed of 21 items that encompass cognitive, affective, behavioral, and somatic components of depression. BDI individual questions evaluate mood, pessimism, sense of failure, self-dissatisfaction, guilt, punishment, self-dislike, self-accusation, suicidal ideas, crying, irritability, social withdrawal, body image change, work difficulty, insomnia, fatigability, loss of appetite, weight loss, somatic preoccupation, and loss of libido. Items 1 to 13 evaluate psychological symptoms, while items 14 to 21 evaluate physical symptoms. Each item offers four statements that vary as to intensity (0 to 3), and respondents should point out which of the four best describes their symptoms. The final score is the sum of the 21 items and yields a four-factor ranking: no depression, mild-moderate depression, moderate to severe depression, or severe depression. The BDI-II items are consistent with the DSM-IV criteria for diagnosis of depressive disorders (Beck & Steer, 1996).

#### FPI

The Fertility Problem Inventory aims to measure topics related to social concern, sexual concern, relationship concern, rejection of childfree lifestyle, and need for parenthood, based on a 46-item scale; it has been validated for Brazil (Ribeiro, 2007). In this study, the FPI was answered individually and scored as to agreement or disagreement about each statement on a six-point Likert scale. The sum of items establishes four factors: social relationship concerns, sexual relationship concerns, rejection of childfree lifestyle, and need for parenthood (Newton *et al*., 1999; Gourounti *et al*., 2010; 2016).

### Statistical analysis

Categorical variables were described as absolute and relative frequencies, and quantitative variables, as means and standard deviations. Normality of continuous variables was verified through the Shapiro-Wilk test. FertiQoL scores were compared with clinical and sociodemographic variables using Student’s t-test and analysis of variance (ANOVA). Variables with *p*-values < 0.20 were included in the multivariate linear regression model to check for independent association with FertiQoL. Associations between FPI and FertiQoL scores were assessed through the Pearson correlation coefficient. All analyses were performed in PASW Statistics, Version 18.0 (SPSS Inc.).

## RESULTS

The sample included 89 participants. Regarding demographic characteristics, 72 (81%) were above age 30, 75% had completed secondary education, and 77 (86.5%) worked or were otherwise economically active. In relation to clinical characteristics, 84% had never had children, 33% had already had a previous assisted reproduction experience, and in nearly 84% of cases the cause of infertility was on the female side. Thirteen patients (14.6%) had a history of miscarriage, and 22.5% were BDI positive. FPI and FertiQoL scores are shown in Table 1. The mean (SD) overall FertiQoL score was 70.9 ± 15.6.

**Table 1:** Demographic and clinical profile and FPI and FertiQoL scores of the sample.

Variable	N (%)
Age (years) ≤ 30 31 to 35 > 35	17 (19.1) 42 (47.2) 30 (33.7)
Educational attainment Some primary Complete primary Secondary Higher	11 (13.8) 11 (13.8) 38 (47.5) 20 (25.0)
Economically active Yes No	77 (86.5) 12 (13.5)
Previous children No Yes (natural/non-natural)	75 (84.3) 14 (15.7)
Previous assisted reproduction No Yes	59 (67) 29 (33)
Cause of infertility Male Female Both	14 (16.1) 61 (70.1) 12 (13.8)
Assisted reproduction method ICSI IVF	11 (12.4) 78 (87.6)
Previous miscarriage Yes No	13 (14.6) 76 (85.4)
BDI Negative Positive	69 (77.5) 20 (22.5)
Total and subscale scores	Mean (SD)
FPI Social relationships Life without children Sexual and conjugal relationship Maternity/paternity	2.8 (0.99) 2.8 (0.78) 4.5 (0.79) 3.9 (0.76)
FertiQoL Central - Emotional Central - Mind Body Central - Relational Central - Social Central - Total Treatment - Environment Treatment - Tolerability Treatment - Total Total	53.8 (20.8) 66.3 (22.7) 74.8 (17.2) 63.9 (20.7) 64.8 (17.0) 70.0 (20.3) 72.1 (22.3) 70.9 (15.6) 66.5 (14.5)

BDI: Beck Depression Inventory; FPI: Fertility Problem Inventory; ICSI: intracytoplasmic sperm injection; IVF: *in vitro* fertilization.

Means and standard deviations of FertiQoL scores are presented in Table 2, stratified by the clinical and demographic characteristics. Variables which showed statistical significance in these analyses were carried forward to multiple linear regressions (Table 3).

**Table 2:** FertiQoL scores stratified by other sample variables. Mean (standard deviation).

Variable	FertiQoL subscales								
	Core - Emotional	Core - Mind Body	Core - Relational	Core - Social	Treatment -Environment	Treatment Tolerability	Core - Total	Treatment - Total	Total FertiQoL
Age (years) ≤ 30 31 to 35 > 35 *p*^1^	51.0 (14.5) 56.4 (21.0) 51.7 (23.5) 0.529	68.5 (18.4) 68.9 (22.5) 61.4 (24.8) 0.353	81.1 (15.9) 74.1 (17.9) 72.2 (16.5) 0.218	65.7 (19.1) 66.4 (19.9) 59.5 (22.6) 0.355	77.7 (17.6) 67.9 (21.4) 68.6 (19.9) 0.218	79.7 (12.0) 73.0 (22.8) 66.4 (24.9) 0.135	66.6 (13.9) 66.5 (16.5) 61.2 (19.1) 0.375	78.3 (13.3) 70.1 (16.9) 67.9 (13.8) 0.076	70.0 (11.4) 67.7 (14.1) 63.0 (16.2) 0.220
Educational attainment Some primary Complete primary Secondary Higher *p*^1^	48.6 (14.5) 63.0 (22.8) 52.8 (19.1) 50.2 (24.6) 0.338	56.6 (23.3) 77.0 (14.3) 68.1 (19.8) 59.9 (27.9) 0.094	70.0 (18.7) 78.7 (14.9) 79.4 (13.9) 67.4 (17.0) 0.029	51.2 (19.6) 74.6 (18.4) 67.0 (17.2) 54.8 (23.3) 0.006	73.0 (17.7) 69.5 (25.5) 73.1 (20.6) 62.9 (18.9) 0.330	75.2 (18.2) 83.9 (11.3) 75.0 (18.8) 57.2 (28.7) 0.004	56.6 (15.5) 73.3 (14.2) 66.9 (14.3) 58.2 (20.4) 0.026	72.4 (14.1) 74.8 (17.4) 73.8 (14.9) 62.3 (15.9) 0.046	61.6 (11.4) 73.6 (12.3) 68.9 (12.3) 59.1 (17.8) 0.015
Economically active No Yes *p*^2^	51.7 (24.5) 54.1 (20.4) 0.717	61.2 (28.5) 67.1 (21.7) 0.41	74.8 (26.8) 74.8 (15.4) 0.999	71.2 (24.3) 62.8 (20) 0.195	71.2 (22.9) 69.8 (20.1) 0.826	72.2 (32.9) 72 (20.4) 0.977	64.9 (21.9) 64.7 (16.2) 0.982	71.9 (19) 70.8 (15.1) 0.81	66.7 (19.0) 66.5 (13.8) 0.954
Previous children No Yes (natural/non-natural) *p*^2^	52.9 (20.2) 58.3 (24.3) 0.382	65.8 (22.5) 68.7 (23.9) 0.661	74.1 (17.5) 78.6 (15.4) 0.374	62.2 (20.8) 73.2 (18.4) 0.068	70.3 (19.9) 68.6 (23.5) 0.785	72.3 (20.4) 70.5 (31.5) 0.783	63.8 (16.9) 69.7 (17.3) 0.235	71.2 (15) 69.4 (19) 0.701	66.0 (14.3) 69.5 (15.3) 0.411
Previous miscarriage Yes No *p*^2^	49.2 (18.1) 54.6 (21.3) 0.390	60.7 (20.1) 67.3 (23.0) 0.338	75.9 (13.2) 74.6 (17.9) 0.804	58.0 (18.9) 65.0 (20.9) 0.266	73.1 (13.2) 69.5 (21.3) 0.423	59.1 (26.8) 74.3 (20.8) 0.023	60.9 (13.8) 65.4 (17.4) 0.379	67.8 (15.5) 71.4 (15.6) 0.442	62.9 (13.0) 67.2 (14.7) 0.325
Previous assisted reproduction No Yes *p*^2^	53.3 (20.1) 53.8 (22.2) 0.920	65.9 (22.6) 66.3 (23.1) 0.936	73.2 (18.4) 77.5 (14.4) 0.271	64.1 (20.4) 62.8 (21.6) 0.787	72.1 (18.4) 65.1 (23.4) 0.131	74.4 (21.2) 66.6 (23.9) 0.125	64.2 (16.8) 65.2 (17.3) 0.790	73.1 (13.9) 65.8 (17.5) 0.039	66.7 (14.1) 65.3 (15.1) 0.668
Cause of infertility Male Female Both *p*^1^	59.3 (15.8) 52.3 (21.0) 51 (24.3) 0.487	70.8 (24.6) 64.3 (21.8) 68.2 (25.9) 0.596	73.8 (14.5) 75.9 (18.1) 69.4 (16.8) 0.499	65.5 (21.9) 62.6 (20.3) 68.1 (23.5) 0.678	61.0 (24.5) 70.7 (20.0) 77.1 (16.2) 0.124	74.4 (14.3) 71.1 (24.3) 71.4 (20.8) 0.886	67.4 (17.3) 63.8 (16.4) 64.2 (20.8) 0.784	66.2 (15.2) 71.1 (15.7) 74.7 (16.5) 0.379	67.0 (12.2) 65.9 (14.4) 67.2 (18.5) 0.944
Assisted reproduction method ICSI IVF *p*^2^	58.7 (24.7) 53.1 (20.3) 0.405	71.5 (24.9) 65.6 (22.4) 0.418	74.2 (16.0) 74.9 (17.5) 0.910	60.4 (26.7) 64.4 (19.9) 0.545	58.6 (27.4) 71.6 (18.8) 0.047	69.7 (23.9) 72.4 (22.2) 0.710	66.4 (21.0) 64.5 (16.5) 0.734	62.8 (18.7) 72.1 (14.9) 0.066	65.3 (15.6) 66.7 (14.4) 0.757
BDI Negative Positive *p*^2^	56.5 (21.0) 44.5 (17.6) 0.023	72.2 (18.5) 45.8 (24.2) < 0.001	78.4 (13.4) 62.5 (22.9) 0.007	66.4 (18.9) 55.5 (24.6) 0.038	69.9 (21.9) 70.3 (13.9) 0.923	75.8 (19.4) 59.2 (26.9) 0.016	68.4 (14.8) 52.2 (18.3) <0.001	71.9 (15.9) 67.4 (14.2) 0.250	69.5 (12.5) 56.3 (16.4) <0.001

1 Analysis of variance.

2 Student’s t-test.

**Table 3:** Multiple linear regressions of factors associated with FertiQoL scores.

FertiQoL subscale									
	**Core -** **Emotional**	**Core -** **Mind Body**	**Core -** **Relational**	**Core -** **Social**	**Treatment -Environment**	**Treatment** **Tolerability**	**Core -** **Total**	**Treatment - Total**	**Total** **FertiQoL**
BDI	**-11.9 (-22.2;-1.7)**	**-23.1 (-33.4;-12.8)**	-**12.4 (-20.1;-4.8)**	-7.9 (-17.9;2.0)		**-10.9 (-21.2;-0.7)**	-13.4 (-21.3;-5.4)		**-10.7 (-17.5;-3.8)**
Education Some primary Complete primary Secondary Higher		-0.7 (-15;13.7) **15.5 (1.2;29.8)** 6.7 (-3.9;17.2) ref	4.0 (-6.7;14.7) 10.4 (-0.2;21.1) **11.2 (3.3;19.0)** ref	-2.7 (-16.6;11.2) **19.2 (5.4;33.1)** **11.7 (1.5;21.9)** ref		**19.3 (4.2;34.4)** **24.9 (10.6;39.1)** **18.4 (7.7;29.1)** ref	-0.04 (-11.1;11) **14.2 (3.2;25.2)** 7.8 (-0.3;15.9) ref	6.9 (-4.7;18.5) **11.4 (0.6;22.3)** **11.0 (2.8;19.1)** ref	3.6 (-5.8;13.1) **13.8 (4.3;23.2)** **9.1 (2.1;16.0)** ref
ICSI					-10.9 (-23.9;2)			-5.6 (-15.0;3.9)	
First attempt					5.1 (-4;14.2)	4.0 (-5.6;13.6)		4.5 (-2.8;11.9)	
Miscarriage						-10.6 (-22.8;1.5)			
Age ≤ 30 31 to 35 > 35						6.7 (-6.1;19.5) 8.0 (-2.3;18.4) ref		7.2 (-2.5;16.8) 1.2 (-6.5;8.9)	

Unstandardized regression coefficients, 95% confidence interval. Significant associations are highlighted in bold (*p*<0.05). ref: category to which others (of the same variable) were compared.

BDI and educational attainment were the only factors that remained associated with FertiQoL subscales (except the Environment subscale of the Treatment module) and total FertiQoL score. BDI correlation coefficients ranged from -10.7 (95CI%: -17.5;-3.8) for total score to -23.1 (95CI%: -33.4;-12.8) for the Core - Mind-Body subscore; i.e., on average, patients with BDI-endorsed depressive symptoms scored 11 to 23 points lower on the FertiQoL. Regarding educational attainment, patients with primary or secondary education alone had better quality of life scores, except for the Core - Emotional and Treatment - Environment subscales, when compared to patients with higher education. The difference in scores among patients with different levels of education (primary and secondary vs. higher) ranged from 9.1 to 24.9 points.

The other variables showed no significant correlation with FertiQoL scores.

Table 4 presents correlations between FPI and FertiQoL scores. Except for the Environment subscale of the FertiQoL Treatment module, the social and conjugal/sexual relationships and need for parenthood subscales of the FPI showed significant associations with all FertiQoL subscales as well as with the overall FertiQoL score. The social relationships correlated negatively with FertiQoL subscales, ranging from r=-0.27 (total Treatment module score) to r=-0.82 (total Core module score), suggesting that, the higher the respondent’s social relationships issues, the worse their perceived quality of life. The need for parenthood also presented negative coefficients, ranging from r=-0.30 (total Treatment module score) to r=-0.66 (total Core module score), i.e., the greater the respondent’s desire to become a parent, the worse their perceived quality of life. On the other hand, the sexual and conjugal relationship subscale correlated positively with the FertiQoL subscales, with coefficients ranging from r=0.25 to r=0.70, demonstrating that, the better the respondent’s relationship with her spouse, the better her perceived quality of life. The childfree lifestyle FPI subscale presented only one significant correlation, with the FertiQoL Core - Emotional; the positive coefficient (r=0.31, *p*<0.01) suggests that the lower the respondent’s desire to have children, the better her fertility-related quality of life in this particular aspect.

**Table 4:** Pearson coefficients of correlation between FPI domains and FertiQoL scores.

FPI		Core - Emotional	Core - Mind Body	Core - Relational	Core - Social	Treatment - Environment	Treatment Tolerability	Core - Total	Treatment - Total	Total FertiQoL
Social concerns	r	-0.65	-0.73	-0.53	-0.78	-0.07	-0.45	-0.82	-0.27	-0.77
	*p*	<0.01	<0.01	<0.01	<0.01	0.49	<0.01	<0.01	<0.01	<0.01
Rejection of childfree lifestyle	r	0.31	0.12	0.04	0.17	-0.09	0.08	0.20	-0.05	0.15
	*p*	<0.01	0.25	0.74	0.11	0.39	0.44	0.07	0.65	0.17
Sexual and conjugal concerns	r	0.49	0.63	0.68	0.55	0.03	0.48	0.70	0.25	0.67
	*p*	<0.01	<0.01	<0.01	<0.01	0.79	<0.01	<0.01	0.02	<0.01
Need for parenthood	r	-0.60	-0.60	-0.47	-0.52	-0.10	-0.46	-0.66	-0.30	-0.65
	*p*	<0.01	<0.01	<0.01	<0.01	0.34	<0.01	<0.01	<0.01	<0.01

r: Pearson correlation coefficient; *p: p* -value.

## DISCUSSION

FertiQoL provides a disease-specific, accurate measurement of the impact of infertility on QoL, being a useful tool to assess QoL in infertile couples (Aarts et al., 2011). Our study findings regarding FertiQoL show the same response pattern found for FPI-related findings, corroborating the work of Moura-Ramos et al., (2008; 2011) which investigated the factor structure of the FPI, focusing on its relevance in clinical practice. The FPI reveals the impact of the infertility experience on several domains of people’s lives (social, conjugal, and sexual), as well as the importance of parenthood in the respondent’s life, by evaluating the desire for parenthood and rejection of a childfree lifestyle.

The FPI has been widely used in surveys about experience of infertility (Peterson *et al*., 2003, 2006, 2007; Slade *et al*., 2007; van der Broeck *et al*., 2010; Gourounti, 2010, Ma, F. *et al*., 2018; Donarelli, Z. *et al*., 2018). A survey looking into the factor structure of the FPI confirmed the tool’s original measurement model, but suggested the inclusion of an intermediate conceptual level, namely the evaluation of infertility-related stress, by assessing two conceptual domains: the impact of infertility on the lives of infertile patients and representations of the importance of parenthood. The FPI exhibited invariance of measurement and structure, as well as construct validity, correlating with other measures that assess similar constructs (Moura-Ramos *et al*., 2011; Pedro *et al*., 2019).

There is a relationship between depression and physical health. Depression results from a complex interaction of social, psychological, and biological factors (WHO, 2024). Accordingly, depression is also associated with quality of life. In our sample, the mean total/overall FertiQoL score was 66.5 ± 14.5, and showed a significant association with depression and educational attainment: patients with depression had, on average, a 10.7 (95CI%: -17.5;-3.8) score gap in relation to those who had no depression, while those who had completed only primary or secondary education reported better quality of life compared to those who had completed higher education, with 11.0 (95CI%: 2.84;19.1) and 11.4 (95CI%: 0.6;22.3) point gaps, respectively. This outcome reinforces the bioecological model, whose purpose is to propose scientific bases to inform the design of public policies and social programs that can neutralize disruptive influences on emerging development; this approach, which can be called the *ecology of human development*, is particularly essential for the design of programs meant to promote social or emotional cognitive growth. Assisted reproduction programs require that couples regularly visit a hospital or a doctor’s office; these are ecological transitions as defined by Bronfenbrenner (1996), i.e., a change in setting from a familiar environment to an unknown environment, relating to the set of microsystems a person experiences and the interrelations established within them. In Bronfenbrenner’s model, such ecological transitions refer to the passage from a microsystem to a mesosystem, which is expanded when a person attends a new environment; thus, social and human constructs that operate in diverse settings are interdependent, and they mutually influence one another. Although the brain’s initial development is genetically oriented, it is continually modified, both positively and negatively, by environmental experience. This malleability or modifiability is known as neuroplasticity, and may be an evolutionary mechanism to allow adaptation to changes in the environment (Pascual-Leone *et al*., 2005; Toga *et al.*, 2006). Plasticity enables learning. Individual differences in intelligence may reflect differences in the brain’s ability to develop neural connections in response to experience (Garlick, 2003). Initial experiences may have lasting effects on the nervous central system, learning processes, and information storage (Society for Neuroscience, 2008).

Women affected by secondary infertility scored higher in the Emotional, Mind-Body, and Social domains of the Core FertiQoL module, the Tolerability domain of the Treatment module, and overall QoL (*p* < 0.05). Women seeking psychological support scored lower in all domains, except Environment (Treatment module). Prolonged infertility was associated with lower scores in the Mind-Body, Social, and Tolerability domains, as well as in overall QoL (*p* <0.05). Multiple regression analysis showed educational status, and secondary infertility had a positive impact, while psychological support had a negative impact on total QoL scores. In a previous study, QoL scores ranked better among secondary infertility patients and those with a higher level of education. Scores were negatively affected by prolonged infertility and support seeking (Karabulut, *et al*., 2013). In our sample, QoL scores showed different outcomes in relation to the education variable compared to those reported by Karabulut *et al*., (2013): patients with higher education degrees scored worse in all QoL dimensions, except the treatment environment and emotional dimensions. Indeed, patients who reported higher educational attainment had the highest depression scores.

Developing a deeper understanding of the experience of infertility-that is, understanding the meaning of parenthood and of a childfree life, which can account for the variability found in adjustment of infertile patients (Greil *et al*., 2011; Moura-Ramos *et al*., 2011; Cheng *et al*., 2018)-is truly important.

Multiple recommendations and guidelines are available on the provision of psychosocial care at assisted reproduction centers. Our findings corroborate that infertility is associated with depression and lower quality of life. Therefore, we believe the inclusion of a psychologist with specialized training in assisted reproduction in the treatment team is essential. Psychological assessment could bring to light factors that contraindicate assisted reproductive treatment altogether; these patients could be referred to psychotherapy or to their general practitioner instead, as appropriate (Bogovic *et al*., 2024; Braverman *et al*., 2024; Salari *et al*., 2024).

In Brazil, the National Humanization Policy (*Política Nacional de Humanização*, PNH) must be part of and be taken into account by all policies and programs implemented by the national Unified Health System (*Sistema Único de Saúde*, SUS). Based on the principle of transdisciplinarity, PNH aims to acknowledge the diverse types of health practices and forms of expertise and how they may interact with the experiences of those who receive care. Together, these different fields of knowledge can lead to a more jointly responsible provision of care (PNH, 2013). Considering the psychological characteristics of infertile women undergoing assisted reproductive treatment observed in this study and aiming to improve their quality of life, implementation of the PNH appears particularly important in this setting.

This study has relevant implications for research. The FPI seems to be a suitable tool to measure infertility-related stress, as it provides a comprehensive assessment of stress associated with infertility in several areas. It can also be useful in clinical settings for screening purposes, by identifying particularly troublesome aspects and risk of emotional distress in men and women facing infertility.

Our findings corroborate previous literature which has associated female infertility with depression and lower quality of life (Bhamani et al., 2020), as demonstrated by the significant negative correlation between the Mind-Body FertiQoL subscale and the BDI; participants with BDI scores consistent with depression scored, on average, 13.4 points less on this scale than their peers without depression. It also bears noting that, in our sample, patients with higher educational attainment also had higher depression levels. Assisted reproduction providers should be aware of this phenomenon and of the multiple factors potentially involved, and should offer psychosocial care before, during, and after treatment.

The limitations of this study include that only women were interviewed: future research could include both parental parties. Furthermore, this study took a cross-sectional approach, whereas future studies could employ longitudinal designs to follow treatment outcomes over time and even potentially investigate childcare.

A comprehensive approach to infertility care that incorporates the promotion of emotional health involves challenges, including the rebirth of hope. We suggest that future research could focus on the conjugal relationship within the context of depression and postpartum depression, searching for a possible association in women who have been diagnosed with infertility. Postpartum depression is not always noticed by family members or healthcare professionals; therefore, new studies on the intensity of distress caused by infertility, involving professionals who are in contact with this population and are aware of their needs for psychological assessment, are needed. This would articulate the diverse knowledge compartmentalized within the most varied fields of knowledge without losing sight of the essence and particularity of each phenomenon, reconnecting matter and spirit, nature and culture, subject and object, objectivity and subjectivity.
